# Effects of Early Language Deprivation on Brain Connectivity: Language Pathways in Deaf Native and Late First-Language Learners of American Sign Language

**DOI:** 10.3389/fnhum.2019.00320

**Published:** 2019-09-19

**Authors:** Qi Cheng, Austin Roth, Eric Halgren, Rachel I. Mayberry

**Affiliations:** ^1^Department of Linguistics, University of California, San Diego, San Diego, CA, United States; ^2^Department of Radiology, University of California, San Diego, San Diego, CA, United States

**Keywords:** deaf, sign language, language deprivation, white matter pathway, diffusion tensor imaging

## Abstract

Previous research has identified ventral and dorsal white matter tracts as being crucial for language processing; their maturation correlates with increased language processing capacity. Unknown is whether the growth or maintenance of these language-relevant pathways is shaped by language experience in early life. To investigate the effects of early language deprivation and the sensory-motor modality of language on white matter tracts, we examined the white matter connectivity of language-relevant pathways in congenitally deaf people with or without early access to language. We acquired diffusion tensor imaging (DTI) data from two groups of individuals who experienced language from birth, twelve deaf native signers of American Sign Language, and twelve hearing L2 signers of ASL (native English speakers), and from three, well-studied individual cases who experienced minimal language during childhood. The results indicate that the sensory-motor modality of early language experience does not affect the white matter microstructure between crucial language regions. Both groups with early language experience, deaf and hearing, show leftward laterality in the two language-related tracts. However, all three cases with early language deprivation showed altered white matter microstructure, especially in the left dorsal arcuate fasciculus (AF) pathway.

## Introduction

Human language is a highly complex cognitive system that relies on a distributed neural network. One crucial question regarding the neurobiology of human language is the role of language experience during development. Early neural plasticity allows environmental experience and learning to shape postnatal brain development (Huttenlocher, 2002) and is often limited to a critical period (Hensch, 2005). Although a similar critical period has been suggested for language development (Lenneberg et al., 1967), it remains unclear how experience within a critical time window contributes to language acquisition. The question is difficult to investigate because neural changes at different levels occur simultaneously during the first few years of postnatal development in typically developing children.

One approach to the question is to compare populations with different early language experience. Although spoken language is ubiquitous for children who hear normally, congenitally deaf children do not have access to it from birth. Approximately 10% of the deaf children are born into deaf families who use sign language as their main communication method. Sign languages are natural languages with linguistic features similar to spoken languages (Stokoe, 1978; Klima and Bellugi, 1979), and the developmental milestones for sign language are similar to those of spoken languages (Reilly et al., 1990; Anderson and Reilly, 2002; Pichler, 2002; Mayberry and Squires, 2006). Deaf children with deaf parents who sign with them thus experience language from birth, like typically developing children with normal hearing. But their early language experience occurs via the visual-manual modality in contrast to the auditory-oral modality of hearing children’s language experience. However, the majority of deaf children are born into hearing families. Some families learn sign language to communicate with their deaf children. Some families prefer to use oral communication with their deaf children, often using hearing compensation technologies such as cochlear implants or hearing aids, together with speech training. Under certain conditions, neither spoken language nor sign language is accessible to deaf children resulting in early language deprivation. Thus, congenitally deaf individuals vary in their early language environment, offering a rare opportunity to investigate the role of early language experience in the development of the neural language network. Research on this question is also crucial to raise the awareness of the potential negative sequelae of early language deprivation in deaf children.

Recent neurolinguistics models (Hickok and Poeppel, 2004, 2007; Parker and Brorson, 2005; Saur et al., 2008; Friederici, 2009; Price, 2012) have identified two information streams, the dorsal and ventral streams, as being crucial for maintaining the dynamic language network. The dorsal stream involves the temporal-parietal-frontal connections mainly via the superior longitudinal fasciculus (SLF) - arcuate fasciculus (AF) complex (Catani and Mesulam, 2008). The ventral stream runs through the extreme capsule (EmC) linking middle-posterior STG to the anterior IFG; the inferior fronto-occipital fasciculus (IFOF) that establishes the occipital-temporo-frontal connection; the inferior longitudinal fasciculus (ILF) connecting the occipital lobe and the temporal lobe; and the uncinate fasciculus (UF) connecting anterior temporal to inferior frontal areas (see Dick and Tremblay, 2012, for a review on the anatomy and functions of each fiber tract).

The dorsal stream is often considered to be responsible for an auditory–motor integration function, carrying acoustic speech signals from the auditory cortex to articulatory representations in the frontal lobe, while the ventral stream is more responsible for speech recognition, and involves structures in the superior and middle temporal lobe that are crucial for meaning and comprehension (Hickok and Poeppel, 2004, 2007; Saur et al., 2008). The dorsal stream has been identified in several studies as also being relevant to complex syntactic processing (Caplan et al., 1986; Wilson et al., 2011; Verhoeven et al., 2012; Meyer et al., 2014; Skeide et al., 2016). It is unclear if deficits at the syntactic level of language are secondary to deficits in other lower-level functions mediated by the dorsal pathways, such as auditory-motor integration and working memory. In addition, previous studies have consistently found a left-ward lateralization pattern, with greater volume, more streamlines, and greater microstructural integrity in the left AF compared to the right AF (Büchel et al., 2004; Catani et al., 2007; Glasser and Rilling, 2008; Ocklenburg et al., 2013; Takao et al., 2013; Eichert et al., 2018). This lateralization pattern is found in children and adolescents as well as in adults (Lebel and Beaulieu, 2009). Eichert et al. (2018) compared the laterality of dorsal AF with the ventral IFOF in humans and macaque monkeys. In humans, the dorsal AF but not the ventral IFOF pathway is left-lateralized, while neither tract is lateralized in macaques. Panesar et al. (2018) also found strong left-lateralized connectivity patterns for ILF in humans. Variability is observed in the exact laterality of UF across studies (Malykhin et al., 2008; Hasan et al., 2009; Yasmin et al., 2009; Danielian et al., 2010; Jahanshad et al., 2010; Ocklenburg et al., 2013; Takao et al., 2013). Less information on laterality is available for EmC.

These language-related pathways mature relatively late in development (Lebel et al., 2008, 2012; Brauer et al., 2011; Perani et al., 2011), and their degree of maturation correlates with language development. Compared to sensorimotor regions, language-related temporal and frontal regions in the left hemisphere show protracted myelination development (Pujol et al., 2006). Accelerated vocabulary development after the age of 18 months relates to a rapid myelination phase in the language-related regions. Children aged 5 to 9 years show a delayed gray matter thinning process in left IFG (Broca’s area) and bilateral posterior temporal regions (Sowell et al., 2004). At age 7 children show immature AF-SLF and IFOF pathways compared to adults (Brauer et al., 2013), and the degree of maturation of the AF-SLF pathway from the ages of 3 to 10 years correlates with children’s comprehension of complex sentences (Skeide et al., 2016). The protracted maturation of the language-related pathways might indicate an extended plastic period that can be shaped by language in the environment.

The majority of studies on language pathways have been conducted with hearing individuals who use spoken languages. To date few studies have directly examined the white matter pathways for sign language in deaf native signers. An empirical question is whether the sensory-motor modality of language affects the connectivity of the language network.

Existing studies on white matter connectivity in congenitally deaf individuals have generally found decreased white matter volume or altered white matter microstructure mostly restricted to auditory-related areas, such as bilateral Heschl’s gyrus (HG), planum temporale (PT), and STG, but not in long range language pathways (Emmorey et al., 2003; Li et al., 2012; Hribar et al., 2014; Karns et al., 2017). One study comparing white matter microstructure in deaf and hearing individuals found additional differences in several language-relevant fiber tracts, such as bilateral SLF and left IFOF (Kim et al., 2009). One factor that may account for the inconsistencies across studies is variation in the developmental onset of language experience among deaf individuals. Because Kim and his collaborators did not report the language acquisition backgrounds of their deaf participants, it is possible that the differences they observed between the deaf and hearing participants were due to effects related to early language deprivation. Deaf individuals who first acquire language later in life show low levels of language proficiency across levels of linguistic structure compared to deaf individuals who experience language from birth (Newport, 1990; Mayberry and Eichen, 1991; Mayberry and Lock, 2003; Boudreault and Mayberry, 2006; Mayberry et al., 2017). Deaf individuals who experienced language deprivation also show altered neural activation patterns compared to deaf individuals with typical language development (Mayberry et al., 2011, 2018; Ferjan Ramirez et al., 2014, 2016), for both lexical and sentence processing. Still, little is known about how early linguistic experience affects the connections between crucial language regions.

In the present paper, we investigate two contrasting factors in early language experience, namely, the sensory-motor modality of early linguistic experience, and the presence/absence of early linguistic exposure. We report the results of a diffusion tensor imaging (DTI) study with 12 hearing native speakers of English who were L2 learners of ASL, 12 deaf native signers of ASL who were L2 learners of English (mostly in the written form), and 3 deaf individuals who experienced extreme language deprivation throughout childhood and who experienced ASL as their first language in adolescence or early twenties.

There are two alternative hypotheses for the effects of the sensory-motor modality of language. One possibility is that deaf native signers establish both dorsal and ventral neural pathways for language processing, with the ultimate size and strength being indistinguishable from that of hearing speakers. Despite the modality difference, the neural correlates for sign language processing are very similar to those for spoken language processing. Lesion studies show that left perisylvian regions are required for sign language use (Hickok et al., 1998; Atkinson et al., 2005). In addition, neural imaging studies show that sign language tasks also activate fronto-temporal regions, especially the left IFG and the left posterior superior temporal lobe (Petitto et al., 2000; MacSweeney et al., 2002; Sakai et al., 2005; Mayberry et al., 2011; Leonard et al., 2012), which is similar to the language network reported for spoken language. Thus, it is likely that dorsal and ventral language pathways connecting IFG and the temporal cortex are also crucial for sign language processing. Alternatively, since sign languages use the visual modality to process linguistic information, there might be structural plasticity of language-related white matter connectivity, which may yield alternative pathways for sign language processing. If so, we would expect deaf native signers to show less robust connectivity for those pathways crucial for spoken language processing, such as AF-SLF and UF, but potentially more robust connectivity for pathways that link the visual cortex and the language regions, such as ILF and IFOF.

For the effects of early language experience, given that late L1 learners show deficits in morpho-syntactically complex structures (Newport, 1990; Mayberry and Eichen, 1991; Mayberry and Lock, 2003; Boudreault and Mayberry, 2006), we might expect to find main differences to be located within the dorsal pathways that are thought to be crucial for complex sentence development and processing. Alternatively, it is possible that both ventral and dorsal pathways are affected, considering the findings from Kim et al. (2009). Another less likely possibility is that development of the language pathways is solely biologically predetermined and unaffected by early language experience. If so, we would not observe any differences between late L1 learners and deaf native signers in terms of white matter connectivity.

## Materials and Methods

### Participants

Twenty-seven adults participated in the study. The protocol was approved by the Institutional Review Board (IRB) of the University of California San Diego.

Two groups of deaf and hearing individuals with robust early language experience were scanned to examine the effects of sensory-motor modality in their early language experience. The group of deaf native signers consisted of twelve participants who were all born severely to profoundly deaf and acquired ASL as their first language from birth from their deaf parents (Table 1). The group of hearing participants consisted of twelve participants who were native English speakers and had taken 40 to 50 weeks of college-level ASL instruction (Table 1). All participants were right-handed adults with no history of neurological or psychological impairment. The hearing L2 signers speakers serve as a sensory-motor modality contrast for the deaf native signers. Like the native signers, they experienced language from birth albeit in the auditory-vocal modality instead of the visual-manual one. Because previous research has consistently shown insignificant white matter changes during young adulthood (Good et al., 2001; Brickman et al., 2006; Lebel and Beaulieu, 2011), we did not strictly control for age in these two groups. We compare the deaf native signers to the hearing L2 ASL signers, instead of monolingual English speakers, because the deaf native signers are all also bilingual in ASL and English (mostly in the written form).

**TABLE 1:** Summary of demographic information for each group.

**Group**	**Number^1^**	**Age (sd)**	**L1 Modality**	**L1 Onset**	**L1 Duration (years)**
Deaf native signers	12 (5)	33.33 (4.1)	Visuo-manual	Birth	Same as age
Hearing L2 signers	12 (11)	24.2 (3.9)	Auditory-oral	Birth	Same as age
Deaf late signers	Carlos	16	Visuo-manual	13	3
	Shawna	16	Visuo-manual	14	2
	Martin	51	Visuo-manual	21	30

*^1^Total number of participants in each group (female participants in the parentheses). For the deaf late signers, each special case is listed by pseudonym.*

Three individuals also participated in the current study as special cases, allowing us to examine how the presence/absence of early language experience affects the language pathways. These individuals were born deaf and experienced severe language deprivation throughout childhood. Their pseudonyms are Carlos, Shawna, and Martin. Due to various circumstances, these otherwise healthy deaf individuals were mainly raised at home with hearing, non-signing family members during childhood and so acquired neither spoken nor sign language and were illiterate. Carlos and Shawna began learning ASL at the age of 13 and 14, respectively, when they were immersed in the same sign language environment for the first time. They had fewer years of ASL exposure at the time of testing compared with Martin who began learning ASL in his 20 s and had 30 years of ASL experience at the time of testing.

Carlos was born into a hearing and non-signing family in another country and received no special services for deaf children, including schooling. He immigrated with family members to the United States at age 11. He was placed into a group home for deaf teenagers at age 13 years and 8 months, which was his first exposure to American Sign Language (ASL). At the time of scanning, he was 16 years and 10 months old, with 3 years and 2 months of daily exposure to ASL.

Shawna was raised by hearing and non-signing guardians and kept at home until the age of 12. She sporadically attended several schools, both deaf and mainstream, for a total of 16 months. At the age of 14 years and 7 months, she was placed into the same group home as Carlos, which marked her first language immersion experience. Shawna was 16 years and 9 months old at the time of scanning, with 2 years and 2 months of daily exposure to ASL.

Martin was born into a hearing and non-signing family in rural Mexico and attended no school until age of 21 when he learned some Mexican Sign Language at a school for deaf children. He immigrated to the United States at age 23, where he learned ASL. Since then, he has used ASL daily with deaf signers, including his wife, co-workers, and friends. At the time of scanning, he was 51 years old, with 30 years of sign language experience.

Elsewhere we have reported in detail on the language development and neurolinguistic processing of these case studies. Despite wide variation in their early home environments, these three cases of childhood language deprivation showed similar patterns of ASL acquisition (Ramírez et al., 2013; Cheng and Mayberry, 2019). They can comprehend some basic syntactic structures but show difficulties with morpho-syntax and complex sentence structures. Their neural activation patterns in response to single ASL signs primed with pictures was imaged with anatomically constrained Magnetoencephalography (aMEG). All three cases showed atypical localization patterns for single signs in comparison to deaf native and hearing L2 signers (Ferjan Ramirez et al., 2014, 2016; Mayberry et al., 2018).

Table 1 summarizes the demographic information of the deaf native signer group, the hearing L2 group, and each deaf individual with delayed L1 onset.

### Image Acquisition

MRI scans were performed at the UCSD Radiology Imaging Laboratory on a General Electric 1.5 Tesla EXCITE HD scanner with an eight-channel phased-array head coil. Four scans were conducted, including one conventional three-plane localizer, one T1 weighted anatomical scan using IR-SPGR sequence with prospective motion (PROMO) correction, one diffusion-weighted scan using single-shot echo-planar sequence with isotropic 2.5 mm voxels and 30 diffusion gradient directions using *b*-value of 1000 s/mm2 (TE/TR 80.4 ms/14,300 ms), and one non-diffusion-weighted (T2) scan using fast spin echo sequence with prospective motion correction.

### Image Processing and Fiber Tracking

For preprocessing, T1-weighted images were corrected for non-linear warping (Jovicich et al., 2006) and spatial sensitivity inhomogeneities (Hagler et al., 2009) using customized processing stream written in MATLAB. As for the diffusion-weighted images, we performed four pre-processing steps, including motion correction (Hagler et al., 2009), eddy current correction (Zhuang et al., 2006), b0 distortion correction (Chang et al., 2012), and gradient non-linearity correction (Jovicich et al., 2006).

We fit the diffusion tensors (DTs) and diagonalized the DT matrices using singular value decomposition to obtain three eigenvectors and their corresponding eigenvalues. We then calculated the fractional anisotropy (FA) ratio from the eigenvalues.

We used a probabilistic tract atlas (Hagler et al., 2009) to identify tracts of interest. We chose to use a probabilistic fiber tracking method instead of a deterministic method because it can better handle the problem of crossing fibers and stray fibers and avoids the subjectivity involved in manually selecting ROI seeds. This atlas has been used across different populations including healthy adults with an age range of 21 to 80 (Perry et al., 2009), epilepsy patients (Hagler et al., 2009), young children and adolescents with an age range of 3 to 20 (Jernigan et al., 2016), and typical and autistic toddlers with an age range of 1 to 4 years (Solso et al., 2016).

The atlas used manually identified three-dimensional maps of streamline fiber counts in 42 individuals together with their T1-weighted images to create co-registered, normalized, average fiber density maps, which provide probabilistic information about the locations and orientations of 23 fiber tracts. Fiber tracts were first manually identified for each individual in DTI Studio (Mori et al., 2005) using multiple ROIs to select a population of streamlines that followed the paths known from anatomy (Wakana et al., 2004), mainly following a 2-ROI approach with the addition of subsequent multiple ‘NOT’ ROIs to remove extraneous fibers that are not a part of the pathway. The goal is to reliably reconstruct fiber bundles that are anatomically accurate with a focus on the core of the fiber bundle. Next, normalized and averaged three-dimensional maps of streamline fiber counts were generated as fiber density maps and co-registered with the common T1 space. Cross-subject average tensors were calculated to provide information about the range of possible diffusion orientations at each location in atlas space. The atlas therefore provides probabilistic information about the locations and orientations of each fiber tract.

Based on previous studies (Hickok and Poeppel, 2004, 2007; Parker and Brorson, 2005; Saur et al., 2008; Friederici, 2009; Dick and Tremblay, 2012; Price, 2012), we selected four fiber tracts from the atlas as relevant long-range pathways for language, namely AF (the direct long segment of the SLF-AF complex), IFOF, ILF, and UF.

So far there is no consensus on the anatomical classification of the SLF-AF complex, and different subcomponents have been proposed, but their functions are still under debate. In the current study, we only looked at the long segment that directly connects the frontal and the temporal regions, as described in the delineation methods, because this is the classic dorsal AF pathway that has been extensively studied in terms of anatomical structures and functions.

One ventral pathway, EmC, was not included here. There are limited anatomical studies on the human EmC, and it is difficult to reliably reconstruct this fiber tract and delineate it from neighboring tracts such as the external capsule, IFOF, and UF. This pathway is not available in most major DTI atlases due to a lack of reliable means to identify it using the 2-ROI approach. Therefore, we omitted this pathway in the current study to ensure that we could delineate the correct fiber tracts.

Below we describe how the selected fiber tracts were manually delineated in DTI Studio based on the documentation from the probabilistic tract atlas.

The AF was manually delineated by the following steps. First, the most inferior axial slice in which the fornix could be seen as a single structure was identified. On the same axial slice, the anterior-posterior midpoint of the posterior limb of the internal capsule was identified, and a coronal slice at this midpoint was chosen. On this slice, an ‘OR’ ROI was selected by choosing the superolateral area just lateral to the posterior limb of the internal capsule including all superior and lateral gyri coming from this core. Next, the midpoint of the splenium of the corpus callosum in a coronal slice was identified, and an “AND” ROI was drawn around the entire ipsilateral hemisphere. In addition, “NOT” ROIs were selected to avoid fibers extending into the external capsule, fibers extending inferiorly through the brainstem, and fibers extending through the cingulum. Next, the anterior commissure was identified in an axial slice and the visible fibers lateral to the sagittal striatum were selected as an “AND” ROI, while ‘NOT’ ROIs were selected to avoid fibers extending superiorly to the parietal lobe and through the cingulum.

The IFOF was manually delineated by the following steps. First, the anterior-posterior midpoint in a coronal slice between the posterior edge of the splenium of the corpus callosum and the occipital pole was identified. An “OR” ROI was drawn around the occipital lobe, inferior to the parietal-occipital sulcus. Next, the most anterior edge of the genu of the corpus callosum was identified in a coronal slice, and an “AND” ROI was drawn around the entire ipsilateral hemisphere. In addition, “NOT” ROIs were selected to avoid fibers extending superiorly and posteriorly beyond the parietal-occipital sulcus and extending through the thalamus.

The ILF was delineated by the following steps. First, the most posterior coronal slice in which the cingulum was visible was selected, and an ‘OR’ ROI around the entire hemisphere was selected. Next, the most posterior coronal slice in which the temporal lobe was visibly distinct from the frontal lobe was selected, and an “AND” ROI was drawn around the entire temporal lobe. In addition, “NOT” ROIs were selected to avoid fibers extending to the contralateral hemisphere at the mid-sagittal line, fibers extending superiorly to the parietal lobe beyond the parietal-occipital sulcus, and fibers extending anteriorly that terminate in the frontal lobe.

The UF was delineated by the following steps. First, the most posterior coronal slice in which the temporal lobe was visibly distinct from the frontal lobe was selected, and an “OR” ROI was drawn around the entire temporal lobe, and another “AND” ROI was drawn around the external capsule. In addition, “NOT” ROIs were selected to avoid fibers extending posteriorly from the main bundle of the uncinate and short fibers at the temporal stem that did not fully extend into the temporal and frontal lobes.

Using Freesurfer (Dale et al., 1999), we first non-linearly morphed individual T1-weighted images to align with the atlas space using the method of discrete cosine transforms (Friston et al., 1995). Diffusion-weighted images were first rigid-body-registered to corresponding T1-weighted images resampled to atlas space, and then further registered using joint probability density function (JPDF) method (Leventon and Grimson, 1998). Next, *a posteriori* probability of a voxel belonging to a given fiber tract was estimated given the first eigenvector derived from DT calculations together with the location information (i.e., fiber probability given location alone) and the orientation information (i.e., fiber probability given the DT first eigenvector and the atlas average of DTs rotated and warped into single subject space) from the co-registered and normalized fiber density maps. A probability threshold (relative fiber probability >0.08) was applied following Hagler et al. (2009) to derive regions of interests (ROIs) for each target fibers. This threshold was determined in Hagler et al. (2009) by testing a range of thresholds and choosing the threshold that provided the smallest difference in fiber volumes between manually selected and atlas-derived fiber masks across all subjects and fibers. Finally, the weighted averages of FA was calculated for each fiber tract (Hua et al., 2008). More details of this automated white matter tracking method can be found in Hagler et al. (2009).

**FIGURE 1:**
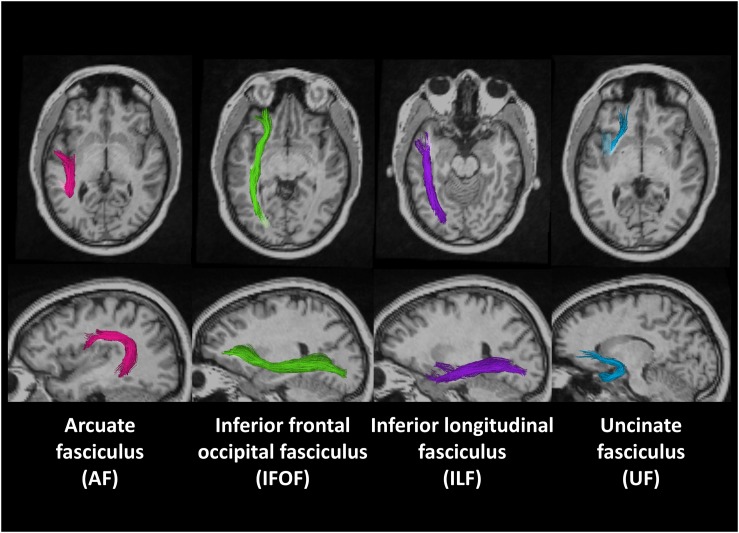
Region of interests (ROIs) of four fiber tracts derived using probabilistic tract atlas in the left hemisphere of one deaf native subject.

Figure 1 shows the locations of these fiber tracts in the left hemisphere found in a deaf native signer participant. We examined these tracts in both right and left hemispheres to examine for possible lateralization effects.

### Statistical Analyses

We used the lme4 package (Bates et al., 2012) in R (R Core Team, 2012) to conduct analyses of variance (ANOVA) tests between the deaf and hearing infant-language experience groups, using the mean FA values of each fiber tract of each individual. We also calculated the z-scores of mean FA for the deaf participants in R.

## Results

### Effects of Early Language Modality

**FIGURE 2:**
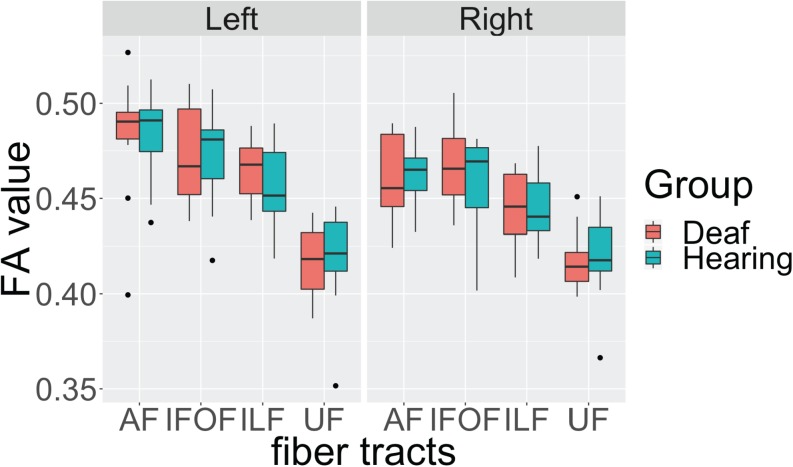
Individual average fractional anisotropy (FA) values of hearing and deaf participants in arcuate fasciculate, AF, inferior frontal occipital fasciculus, IFOF, inferior longitudinal fasciculus, ILF, and uncinate fasciculus, UC, as a function of hemisphere showing no significant differences between the groups. The top of the box plot shows the higher quartile (25%), the black bar shows the median (50%), and the bottom of the box shows the lower quartile (75%); the black dots show outliers outside the 1.75 interquartile range.

First, we investigated the effects of the sensory-motor modality of language by comparing the data from the deaf native signers with that of the hearing native English speakers, L2 signers. The deaf and hearing participants show very similar FA values in all fiber tracts in both hemispheres, with close median values and a similar degree of variance (Figure 2). Also, for both groups the FA values in the left hemisphere appear to be higher than those in the right hemisphere. We conducted an Analysis of Variance (ANOVA) test with FA value as the dependent measure, group as the between-subjects fixed effect, fiber tract and hemisphere as within-subjects fixed effects, and gender and age as between-subjects covariates. After controlling for gender (*F*(1, 174) = 2.596, *p* = 0.108) and age (*F*(1, 174) = 2.924, *p* = 0.089) effects, the results showed a significant difference among fiber tracts (*F*(3, 174) = 60.770, *p* < 0.001), a difference between hemispheres (*F*(1,174) = 14.689, *p* < 0.001) with lower FA in the right hemisphere, a trend toward interaction between fiber tract and hemisphere (*F*(3, 174) = 2.389, *p* = 0.070), but no difference between the groups (*F*(1, 22) = 0.094, *p* = 0.759), and no interactions between group and fiber tract (*F*(3,174) = 0.261, *p* = 0.853), group and hemisphere (*F*(1, 174) = 0.036, *p* = 0.848), or between group, fiber tract and hemisphere (*F*(3,174) = 0.173, *p* = 0.914).

These results indicate that, in general, there is left lateralization of language-relevant tracts in deaf and hearing participants alike, despite differences in the sensory-motor modality of their infant language experience. In addition, the trend for interaction between fiber tract and hemisphere suggests that the degree of lateralization might be slightly different for each fiber tract.

To examine the nature of this trend, we conducted ANOVA tests for each fiber tract with FA as the dependent measure, group as the between-subjects fixed effect, hemisphere as within-subjects fixed effects, and gender and age as between-subjects covariates. We found a strong left lateralization effect for AF (*F*(1, 42) = 10.842, *p* = 0.002) and ILF (*F*(1, 42) = 8.832, *p* = 0.004), but no lateralization effect for IFOF (*F*(1, 42) = 2.210, *p* = 0.144) and UF (*F*(1, 42) = 0.004, *p* = 0.949). Again, no group or interaction effects between group and hemisphere were observed in any of the tracts.

These findings confirm in the present data set that the dorsal AF shows left lateralization reported in the literature. In addition, we found one ventral pathway, ILF, to also show left lateralization, while the other two ventral pathways appear to be more bilateral. Crucially, these lateralization patterns are shared by both deaf and hearing participants, suggesting that language modality is not a factor in these laterality effects.

One observation worth noting is that for the dorsal AF, two individuals from the deaf native signer group and one individual from the hearing speaker group, were below the 1.75 interquartile range of their respective group, but only in the left hemisphere. After examining their individual profiles, we noticed that these individuals all had higher FA values in the right hemisphere for the dorsal AF pathways. According to the literature (Catani et al., 2007; Lebel and Beaulieu, 2009), there are individual differences in the lateralization patterns of this pathway. Therefore, we speculate that these individuals show lower FA in the left hemisphere due to a right lateralization pattern.

### Effects of Early Language Deprivation

Next, we investigated the effects of early language experience on language-relevant fiber tracts by first comparing the data from each of the three deaf case studies who matured without language to the deaf native signers and hearing native speakers who had language experience from birth. We used z-scores and interquartile ranges to estimate the differences between the language deprived cases and the two infant-language experience control groups. We then summarized the similarities and differences across these three cases.

Table 2 shows the z-scores of each deaf case study when calculated within the sample of both deaf native signers and hearing native speakers, 24 individuals in total who experienced language from birth. All three case studies showed decreased FA values in the dorsal AF pathway when compared to the language-from-birth control groups, while their FA values in the ventral pathways showed more individual variation.

**TABLE 2:** *z*-scores of three deaf case studies compared to the infant-language experience groups (*N* = 24).

**Case name**	**AF**		**IFOF**		**ILF**		**UF**	
				
	**Left**	**Right**	**Left**	**Right**	**Left**	**Right**	**Left**	**Right**
Carlos	−1.723^∗^	−2.257^∗^	0.083	–0.634	0.495	0.349	0.432	–0.544
Shawna	–0.903	–0.674	–0.343	0.462	0.735	1.391	1.898^∗^	1.048
Martin	–1.110	–0.825	–0.7	–0.452	–2.65^∗∗^	−1.652^∗^	–0.777	–1.426

*^∗^One-tailed *p*-value < 0.05; ^∗∗^One-tailed *p*-value < 0.01.*

**FIGURE 3:**
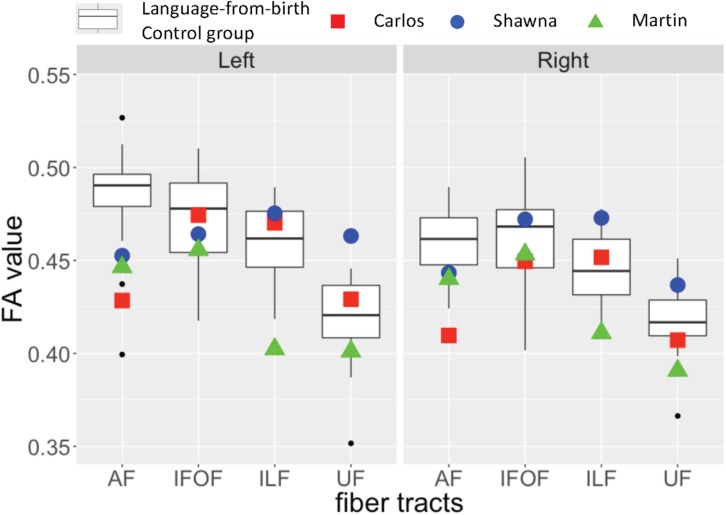
Fractional anisotropy (FA) values for three deaf individuals with severe early language deprivation as a function of pathway and hemisphere. The box plots represent the distribution of FA values of the infant-language experience control groups (both native deaf signers and hearing native English speakers, *N* = 24); the plotted shapes show the values for each late L1 learner: Carlos – filled square; Shawna – filled circle; Martin – filled triangle (Carlos: AoA 13;8 with 3;2 years of experience; Shawna: AoA 14;7 with 2;2 years of experience; Martin: AoA 21 with 30 years of experience). The top of the box plot shows the higher quartile (25%), the black bar shows the median (50%) and the bottom of the box shows the lower quartile (75%); the black dots show outliers outside the 1.75 interquartile range.

Figure 3 shows the FA values of each deaf case in comparison to the interquartile range of the infant-language experience control group, including both deaf native signers and hearing native speakers. The FA values of all three late learners fell outside the infant-language experience control groups’ 1.75 interquartile range for left dorsal AF pathway. Their FA values for AF in the right hemisphere were also lower than the infant-language experience control groups’ interquartile range; only the FA values for Carlos fell outside the 1.75 interquartile range. As for the ventral pathways, the FA values of the case studies were either within normal range or showed more individual variations. Their FA values for bilateral IFOF were all within the interquartile range. For ILF, only the FA values for Martin were below the 1.75 interquartile range in both hemispheres. For UF, Shawna showed an FA value in the left hemisphere above the 1.75 interquartile range. Carlos showed normal FA values in both hemispheres. Martin showed FA values below the interquartile range in the left hemisphere and below the 1.75 interquartile range in the right hemisphere.

As discussed above in section “Effects of early language modality”, some individuals from the infant-language experience control groups also showed decreased FA values in the left dorsal AF due to atypical lateralization patterns. Given that all three late learners showed slightly higher FA values in the left hemisphere for this pathway, and FA values in the right hemisphere that were closer to the interquartile range, we interpret their reduced FA values as revealing a lack of lateralization pattern due to early language deprivation in comparison to the outliers in the infant-language experience control groups who show right lateralization for these tracts.

## Discussion

With respect to the sensory-motor modality of language, we asked if deaf native signers and hearing native speakers would show similar microstructure of the language pathways despite differences in the sensory-motor modality of their early language. We observed no differences between the deaf and hearing groups for any of the four language-relevant pathways. This lack of differences supports the hypothesis that language modality does not affect the connectivity between major language regions when language acquisition begins in infancy.

Effects of neural cross modal plasticity due to sensory (auditory) deprivation among deaf individuals have been reported for some cognitive functions, such as general visuo-spatial working memory (Ding et al., 2015; MacSweeney and Cardin, 2015; Cardin et al., 2018). With respect to language processing when a first language is acquired early on, however, the default language network is unaffected by language modality or sensory (auditory) deprivation (Petitto et al., 2000; MacSweeney et al., 2002; Sakai et al., 2005; Mayberry et al., 2011; Leonard et al., 2012). Our findings provide further evidence for the amodal nature of the language network when infants experience sufficient language during development and further extends them by showing that deafness *per se* does not affect development of the language pathways.

Our findings are also the first to demonstrate left lateralization of two language pathways, dorsal AF and ventral ILF, in deaf native signers, similar to the lateralization that has been consistently found for hearing native speakers (Büchel et al., 2004; Catani et al., 2007; Glasser and Rilling, 2008; Ocklenburg et al., 2013; Takao et al., 2013; Eichert et al., 2018; Panesar et al., 2018). We also found similar bilateral patterns for ventral IFOF and ventral UF in both the deaf and hearing control groups, findings that are also consistent with the literature (Kubicki et al., 2002; Ocklenburg et al., 2013; Takao et al., 2013; Hernando et al., 2015; Eichert et al., 2018).

Crucially, by comparing the data from the language-from-birth control group with that of the three case studies, we found specific effects of early language deprivation on the language pathways. Decreased FA values (below the 1.75 interquartile range) in the left dorsal AF pathway were observed in each of the three cases when compared to the language-from-birth control groups. Given the strong left lateralization patterns observed among the language-from-birth control groups, it appears that the lower FA values in left AF in each case study are due to reduced laterality.

These findings are also consistent with the literature on the relation between the dorsal pathway and the ability to learn and process complex linguistic structures. As explained in the introduction, the dorsal stream is often associated with syntactic processing performance as previously found for typically developing children (Skeide et al., 2016), children with specific language impairment (Verhoeven et al., 2012), and aphasic patients (Wilson et al., 2011). The present study extends these findings to deaf people with early language deprivation, suggesting that their limited syntactic performance may be associated with connectivity deficits in dorsal pathways.

As for the ventral pathways, we observed more individual variations. Martin showed decreased FA values (below the 1.75 interquartile range) in bilateral ILF and in right UF compared to the language-from-birth control groups, while Carlos and Shawna did not show such effects. There are several possibilities for the individual variation in these ventral pathways. One possibility is that the ventral pathways are not as sensitive to a lack of early language experience as the dorsal pathways and remain plastic even after puberty. Martin did not begin to learn language until the age of 21. Carlos and Shawna both began to learn language at the ages of 13 and 14, respectively. In a neuroimaging study of lexico-semantic processing, Carlos and Shawna showed some activation in perisylvian language areas to familiar ASL signs primed with pictures (Ferjan Ramirez et al., 2016). By contrast, Martin showed almost no activation in bilateral temporal language areas, despite performing nearly as accurately and quickly as the deaf native signers, hearing L2 signers, and the other case studies (Mayberry et al., 2018). Thus, it is possible that these individual differences reflect a gradient effect of the duration of language deprivation in childhood and adolescence on the development of the ventral language pathways that potentially mediate lexico-semantic processing. Another possibility for the lack of clear effects of early language deprivation across ventral pathways is that they can be shaped by non-linguistic experience. Because ventral pathways are often associated with constructing meaning, as well as other non-linguistic functions such as complex object processing, late L1 learners may have gradually established these pathways through non-verbal communication and conceptual learning through daily life experience despite the lack of language in the environment. Future studies with a larger sample size and longitudinal observations are required to test these hypotheses.

One caveat in interpreting the present results is that the age of all three late signers was either younger or older than that of the control groups, falling at either the lower or the higher ends of the age range of stable white matter microstructure. However, given the facts that the IFOF often shows a similar trajectory of FA change as a function of age compared to other tracts (Lebel et al., 2008), and that all three cases had normal FA values in bilateral IFOF, we interpret the differences between the cases and the control groups as being more likely due to early language deprivation than simply age.

Another caveat is that Carlos and Shawna only had 2 to 3 years of ASL experience at the time of scanning, so that further neural plasticity induced by late language acquisition might still be possible. We plan to conduct follow-up DTI scans to address this issue. Still, given their limited ultimate attainment of ASL after substantially longer years of exposure, we expect less plasticity in their language pathways even with increased years of language use.

Research has found that deaf children with no formal sign language input often develop their own gestural system to communicate with their family members known as homesign (Goldin-Meadow and Feldman, 1977; Goldin-Meadow and Mylander, 1998). Whether the sophistication of the homesign system the deaf child develops with the family is related to their subsequent sign language development is unknown. Martin reported communicating with a hearing sister with gesture when living at home (Mayberry et al., 2018). Neither Carlos nor Shawna were reported by the knowledgeable professionals working with them to use homesign when they were initially placed in a residential sign language situation (Ferjan Ramirez et al., 2014), but doing so may not have been communicatively useful for them. Although homesign has been found to share some linguistic properties with language (Bates et al., 2012), it has also been observed to be used primarily as an expressive means of communication by the deaf child whose family members may neither fully comprehend nor use it in the same way (Carrigan and Coppola, 2017), thus limiting its potential to circumvent the effects of a lack of linguistic experience for the developing child (Morford and Hänel-Faulhaber, 2011).

The present findings also provide an explanation for some inconsistencies in the literature on white matter connectivity in deaf individuals. Kim et al. (2009) identified more extensive regions with white matter deficits, including non-auditory regions within language-related pathways, while Li et al. (2012) and Karns et al. (2017) found differences only within auditory regions. By explicitly comparing deaf native signers and well-studied cases of extreme delay in the onset of language experience, our findings suggest that the variable results of previous studies are likely due to the diverse language backgrounds that would be characteristic of any randomly selected sample of deaf individuals. Given that Kim et al. (2009) did not report the language backgrounds of their deaf participants, and they also found FA deficits in language-related pathways such as SLF, it is highly probable that their deaf participants experienced language deprivation during childhood, similar to the cases we studied here.

Our findings also shed light on the potential mechanisms of critical period effects for language development. Previous studies have reported selective critical period effects on morphologically and syntactically complex structures (Newport, 1990; Mayberry and Lock, 2003; Boudreault and Mayberry, 2006; Cormier et al., 2012) as well as decreased functional activation in several language regions (Mayberry et al., 2011, 2018; Ferjan Ramirez et al., 2014, 2016). However, it remained unclear how these language and neural outcomes were being influenced by early language experience. The present study shows that the case studies who suffered language deprivation during childhood developed less robust connections between language regions, especially in the dorsal stream. Given the association between dorsal pathways and syntactic processing, a coherent interpretation of the linguistic and neural activation data across these studies is that early language experience is crucial for the growth of the dorsal stream for language processing, linking various functional language regions, and thus facilitating the acquisition and processing of complex syntactic structures. Missing the critical time window for linguistic experience appears to affect development of the dorsal stream, which, in turn, creates deficits in language and neural outcomes, especially with respect to complex morpho-syntactic structures.

To conclude, in the present study we examined white matter microstructure of two groups of individuals with infant language experience, deaf native signers and hearing native speakers who were L2 signers, with three individual cases of childhood language deprivation, individuals who had little access to any kind of language until puberty or after. Our findings indicated that these cases had altered microstructure in some language-related pathways, especially in the left AF, when compared to deaf native signers. At the same time, deaf native signers of ASL showed similar connectivity within language-related pathways compared with hearing native speakers of English. Together these findings suggest that full growth of the brain language pathways requires early language experience during childhood. Language experience in early life appears to be crucial for the language system to become robustly connected as observed in the canonical mature state, regardless of its sensory-motor modality.

## Data Availability

The datasets generated for this study are available on request to the corresponding author.

## Ethics Statement

The studies involving human participants were reviewed and approved by The UCSD Human Research Protections Program (HRPP). The patients/participants provided their written informed consent to participate in this study. Written informed consent was obtained from the individual(s) for the publication of any potentially identifiable images or data included in this article.

## Author Contributions

QC contributed to the study concept and design, data analysis, and wrote the manuscript. AR contributed to the neuroimaging methods and image processing. EH contributed to the study concept and design, data collection, and study supervision. RM contributed to the study concept and design, data collection, study supervision, and revised the manuscript.

## Conflict of Interest Statement

The authors declare that the research was conducted in the absence of any commercial or financial relationships that could be construed as a potential conflict of interest.
